# A case of IgG4-related dacryoadenitis and sialoadenitis remitted by dupilumab monotherapy

**DOI:** 10.1093/rheumatology/kead680

**Published:** 2023-12-13

**Authors:** Ryo Nishioka, Takayoshi Ueno, Dai Inoue, Satoru Kondo, Mitsuhiro Kawano

**Affiliations:** Department of Rheumatology, Kanazawa University Hospital, Ishikawa, Japan; Department of General Medicine, Kanazawa University Hospital, Ishikawa, Japan; Department of Otolaryngology-Head and Neck Surgery, Kanazawa University Hospital, Ishikawa, Japan; Department of Radiology, Kanazawa University Hospital, Ishikawa, Japan; Department of Otolaryngology-Head and Neck Surgery, Kanazawa University Hospital, Ishikawa, Japan; Department of Rheumatology, Kanazawa University Hospital, Ishikawa, Japan

Rheumatology key message

- Dupilumab monotherapy has potential to resolve mass lesions of IgG4-related disease slowly but completely.

Dear Editor, IgG4-related disease (IgG4-RD) is a chronic fibroinflammatory disorder characterized by systemic organ involvement with the infiltration of polyclonal IgG4-positive plasma cells and storiform fibrosis [1]. Although IgG4-RD responds well to glucocorticoid treatment, it often relapses without maintenance therapy, resulting in long-term steroid administration. Long-term exposure to glucocorticoid in IgG4-RD, which commonly develops in elderly people, is a major concern because it increases the risks of various adverse effects including osteoporosis, infection and arteriosclerosis. To reduce exposure to glucocorticoid, addition of various immunosuppressants has been suggested. Dupilumab, a fully human anti-IL-4 receptor α monoclonal antibody, is another choice that some patients with IgG4-RD have been reported to be successfully treated by monotherapy with [2–4]. However, a case of treatment failure with dupilumab has been reported and its efficacy in IgG4-RD has not been established [5]. Furthermore, the mechanism by which dupilumab reduces the mass of IgG4-RD lesions remains unclear. We describe a case of IgG4-RD successfully treated with monotherapy dupilumab, which completely resolved the swollen lacrimal and submandibular glands, and hypothesize the mechanism of dupilumab in IgG4-RD based on recent reports.

A 61-year-old woman presented with the chief complaints of dysosmia, dysacousis, ear fullness, wheezes, swelling of bilateral submandibular glands, and 7 kg weight loss in 4 months. She did not experience fever during this time. Six months prior, she had developed refractory rhinitis, otitis media and bronchial asthma. Physical examination revealed an elastic hard swelling in the bilateral submandibular regions, suggestive of lesions. Ultrasonography showed mild enlargement of bilateral lacrimal glands and apparent enlargement of bilateral submandibular glands. Laboratory tests revealed a white blood cell count of 5370/μl eosinophils comprising 18.0% (normal range 0–4%). CRP was within the normal range at 0.04 mg/dl. Serum levels of IgE were elevated at 427 IU/ml (normal <250 IU/ml), while IgG was in the normal range at 1501 mg/dl (normal 870–1700 mg/dl). IgG4 was elevated at 452 mg/dl (normal 11–121 mg/dl), and antineutrophil cytoplasmic antibody tests were negative. Moreover, fractional exhaled nitric oxide was increased to 246 ppb (normal <37 ppb) despite using inhaled glucocorticoid. A submandibular gland biopsy revealed about 200 IgG4-positive cells per high-power field and an IgG4^+^/IgG cell ratio of 70% leading to the diagnosis of IgG4-RD in accordance with ACR/EULAR classification criteria. Eosinophilic granulomatosis with polyangiitis was ruled out due to the absence of fever, elevated CRP, and signs of vasculitis including purpura, glomerulonephritis, alveolar haemorrhage or mononeuritis multiplex. While the IgG4-RD primarily affected her lacrimal and salivary glands, immediate treatment with glucocorticoid and immunosuppressive agents was not deemed necessary. However, her refractory bronchial asthma required intensified therapy. She declined oral glucocorticoid treatment, so we initiated treatment with dupilumab starting with 600 mg initially and then 300 mg every 2 weeks as a specific treatment for asthma induced by eosinophilic inflammation. With dupilumab treatment, her wheezing symptoms quickly improved, and both the sense of smell and hearing were promptly restored. Furthermore, her enlarged lacrimal glands returned to normal size and her enlarged submandibular glands significantly reduced 2 months after starting treatment, achieving complete resolution 7 months later (Fig. 1). Serum IgG4 level decreased to 205 mg/dl in 2 months and 81 mg/dl in 5 months.

**Figure 1.**
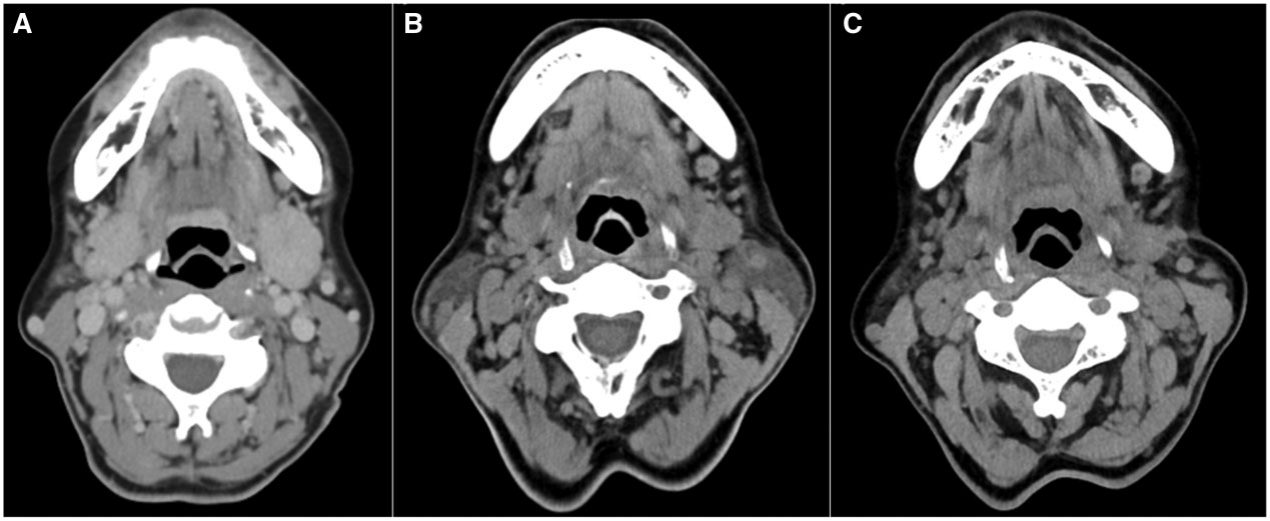
CT findings of submandibular glands before and after dupilumab therapy. CT revealed bilateral enlargement of submandibular glands at first visit (**A**), its reduction at 2 months after initiating dupilumab monotherapy (**B**), and complete resolution 7 months later (**C**)

Our case demonstrated that dupilumab monotherapy has the potential to resolve mass lesions of IgG4-RD slowly but completely. Dupilumab is assumed to inhibit binding of IL-4 to the IL-4 receptor subunit IL-4Rα, recruitment of γc to the IL-4Rα chain and recruitment of IL-4Rα to IL-13Rα1, resulting in blockade of signalling through both type I and type II IL-4R complexes. Because dupilumab facilitates the resolution of mass lesions in IgG4-RD, it could provide insights into how these lesions form. Previous research showed that IL-4-deficient mice have only minimal disruptions in germinal centre formation, suggesting that IL-4 might not be the sole factor responsible for mass lesion formation [6]. However, it has been demonstrated *in vitro* that IL-4 stimulation contributes to shifting the balance of IgG subclasses towards IgG4 by increasing the number of IgG4-positive plasmablasts and plasma cells in IgG4-RD [7]. Considering this, inhibiting the IL-4 pathway with dupilumab might disrupt this balance contributing to the resolution of mass lesions in IgG4-RD by reducing the supply of IgG4-positive cells. Interestingly, Seki, *et al.* reported a case of IgG4-RD where lung lesions spontaneously regressed after excision of submandibular gland lesions [8]. This suggests that various factors, including IL-4, might play a role in sustaining the mass lesions of IgG4-RD.

Although more cases treated with dupilumab monotherapy are required to clarify its mechanism in IgG4-RD, it is important to exercise caution and not apply it specifically in patients with critical organ involvement until its therapeutic effectiveness for IgG4-RD has been firmly established. Currently, it is advisable to consider using dupilumab monotherapy only in patients without life-threatening lesions, similar to our case. By accumulating knowledge from such cases, we may potentially determine the therapeutic effectiveness and mechanism of dupilumab in treating IgG4-RD.

## Data Availability

Raw data were generated at Kanazawa University Hospital. Derived data supporting the findings of this case report are available from corresponding author [M.K.] on request.
